# The Incubation Period of Primary Epstein-Barr Virus Infection: Viral Dynamics and Immunologic Events

**DOI:** 10.1371/journal.ppat.1005286

**Published:** 2015-12-01

**Authors:** Samantha K. Dunmire, Jennifer M. Grimm, David O. Schmeling, Henry H. Balfour, Kristin A. Hogquist

**Affiliations:** 1 Center for Immunology, Department of Laboratory Medicine and Pathology, University of Minnesota Medical School, Minneapolis, Minnesota, United States of America; 2 Department of Pediatrics, University of Minnesota Medical School, Minneapolis, Minnesota, United States of America; Baylor College of Medicine, UNITED STATES

## Abstract

Epstein-Barr virus (EBV) is a human herpesvirus that causes acute infectious mononucleosis and is associated with cancer and autoimmune disease. While many studies have been performed examining acute disease in adults following primary infection, little is known about the virological and immunological events during EBV’s lengthy 6 week incubation period owing to the challenge of collecting samples from this stage of infection. We conducted a prospective study in college students with special emphasis on frequent screening to capture blood and oral wash samples during the incubation period. Here we describe the viral dissemination and immune response in the 6 weeks prior to onset of acute infectious mononucleosis symptoms. While virus is presumed to be present in the oral cavity from time of transmission, we did not detect viral genomes in the oral wash until one week before symptom onset, at which time viral genomes were present in high copy numbers, suggesting loss of initial viral replication control. In contrast, using a sensitive nested PCR method, we detected viral genomes at low levels in blood about 3 weeks before symptoms. However, high levels of EBV in the blood were only observed close to symptom onset–coincident with or just after increased viral detection in the oral cavity. These data imply that B cells are the major reservoir of virus in the oral cavity prior to infectious mononucleosis. The early presence of viral genomes in the blood, even at low levels, correlated with a striking decrease in the number of circulating plasmacytoid dendritic cells well before symptom onset, which remained depressed throughout convalescence. On the other hand, natural killer cells expanded only after symptom onset. Likewise, CD4+ Foxp3+ regulatory T cells decreased two fold, but only after symptom onset. We observed no substantial virus specific CD8 T cell expansion during the incubation period, although polyclonal CD8 activation was detected in concert with viral genomes increasing in the blood and oral cavity, possibly due to a systemic type I interferon response. This study provides the first description of events during the incubation period of natural EBV infection in humans and definitive data upon which to formulate theories of viral control and disease pathogenesis.

## Author Summary

Chronic viral infections are ubiquitous in the human population. Many of these viruses persist in spite of the host immune response. Epstein-Barr virus (EBV) is a human herpesvirus and the primary causative agent of acute infectious mononucleosis. The virus is primarily transmitted through salivary exchange yet the kinetics of dissemination and initial immune response remain poorly understood, especially during EBV’s lengthy six-week incubation period. By doing prospective analysis of natural infection in human subjects, we were able to examine responses during the incubation period. We found that virus disseminates into the blood from the oral cavity much earlier than previously predicted and often before large-scale viral replication in oral cells. This correlated with a systemic innate immune response in the form of type I interferon signaling. A subsequent decrease in circulating plasmacytoid dendritic cells was observed simultaneously with polyclonal CD8 T cell activation. These data suggest that EBV replication is self-limiting in the oral cavity and that infection is established for several weeks before virally infected cells traffic to peripheral blood and initiate innate and adaptive immune response.

## Introduction

Epstein-Barr virus (EBV) is a ubiquitous human herpesvirus. As with all herpesviruses, EBV causes lifelong infection in its host. Infection is associated with autoimmune diseases [1, 2] and is known to cause several types of cancer, representing 1% of the worldwide cancer burden [3]. Primary infection in children is either asymptomatic or causes mild symptoms not readily associated with EBV. In adolescents and young adults, however, EBV is most commonly recognized as the etiologic agent of acute infectious mononucleosis (AIM) [4]. The virus is chiefly transmitted from person to person in oral secretions, although it can be acquired from blood transfusions or from receipt of allogeneic donor cells or tissue. There is currently no vaccine or effective treatment for AIM or other EBV related diseases. Because EBV infection is limited to primates, there are no small animal models of infection except humanized mice [5]. Neither humanized mice nor mice infected with the related gamma herpesvirus MHV68 exhibit true AIM. Therefore a detailed and accurate understanding of primary infection in humans is critical for developing therapeutic tools to treat EBV related diseases.

Abundant data are available on AIM in humans, especially the most severe cases, as subjects are typically seen in clinic. Indeed, the antibody and cell mediated adaptive immune response to the virus, and how it wanes and changes after AIM presents is well established [6]. However a particular knowledge gap exists regarding the events that occur between transmission and symptom onset—the incubation period—which is unusually long, about six weeks [7, 8]. In comparison, most other acute viral infections have incubation periods ranging from less than a day to a week [9]. Thus in particular, we lack knowledge about initial infection events and the innate immune response to EBV in humans; although these are presumed to be critical as EBV has a multitude of innate immune evasion mechanisms [10, 11].

From in vitro studies, we know that EBV efficiently infects B cells through binding of viral gp350 and gp42 proteins to the B cell surface molecules CD21 and HLA Class II, respectively [12]. However, EBV can also infect oral epithelial cells, albeit much less efficiently [13]. It is unknown which cells are initially infected in the oral cavity during natural infection. One possibility is that the virus infects and replicates in oral epithelial cells early during primary infection; but AIM does not occur until B cells are later infected in the tonsils, and virus disseminates to the blood. The virally encoded LMP1 and LMP2 proteins are known to drive infected B cells to differentiate by acting as functional homologues of CD40 and the BCR respectively, and this also triggers migration from tonsils to the blood [14]. Another model proposes that both cell types are infected early in the oral cavity, and that cycles of infection and reactivation must occur during the incubation period to ultimately produce high levels of infected B cells in circulation, which drives AIM [15]. Alternatively, a third model is that B cells may be initially infected in tonsils where they vertically transmit virus at low levels [16]. Infection would be limited to B cells in the nasopharyngeal secondary lymphoid tissue until some stochastic event caused reactivation of the virus, spreading virus to epithelial cells and resulting in an acute increase in viral load, occurring weeks after initial infection. The dynamics of tissue tropism of EBV are interesting in this context, as the virus produced by epithelial cells is particularly efficient at infecting B cells, and vice versa [13]. Each of these three models make distinct predictions about the relative levels of virus in the oral cavity versus blood during the incubation period, and would suggest different strategies to combat infection therapeutically, as antiviral compounds only target actively replicating virus.

Although it is critical to understand the viral and immunological events that occur during the incubation period, it is challenging to obtain both samples and comprehensive clinical data during this period. To address this need, we enrolled undergraduate volunteers who were naïve to EBV and monitored them routinely for natural infection, capturing timepoints within the incubation period by chance. Through frequent sampling, we were able to obtain 48 incubational samples from 40 young adult study participants. We sought to detect and quantify virus in the blood and oral cavity, with particular attention to when virus disseminates from nasopharyngeal tissue to the periphery. In addition, innate and adaptive immune responses during this period were examined, with particular emphasis on natural killer (NK) cells, plasmacytoid dendrtitic cells (pDC), CD8 T cells, and Foxp3+ T regulatory (T_reg_) cells. Surprisingly, during the incubation period, viral genomes were detected at low levels in peripheral blood prior to detection in the oral cavity. A dramatic reduction in blood pDC numbers and a type I interferon response were observed roughly coincident with viral increases in the blood. In contrast, the dramatic increase in CD8 T cell numbers characteristic of AIM, the distinctive type II interferon/cell cycle gene expression signature of AIM, and changes in NK cell phenotype and T_reg_ cell numbers previously reported to occur during AIM were not observed until symptom onset. We discuss possible mechanisms to explain these changes and implications for the treatment of EBV related diseases.

## Results

### Study design

We previously described a prospective study of primary EBV infection in 66 undergraduates at the University of Minnesota [17], 59 of whom were symptomatic. As an extension of this study, we enrolled a new prospective cohort with the specific aim of more frequent sampling in order to serendipitously capture more samples from the incubation period of primary EBV infection. From both cohorts combined, a total of 48 blood and oral wash samples from 40 subjects were obtained during the historically defined incubation period (42 days). These samples were the focus of this study. Since the exact date of infection with EBV is undefinable in a study of natural infection, we designated the date of symptom onset as day “zero.”

### Viral dissemination into circulation occurred before large quantities of viral genomes were detected in the oral cavity

**Fig 1:**
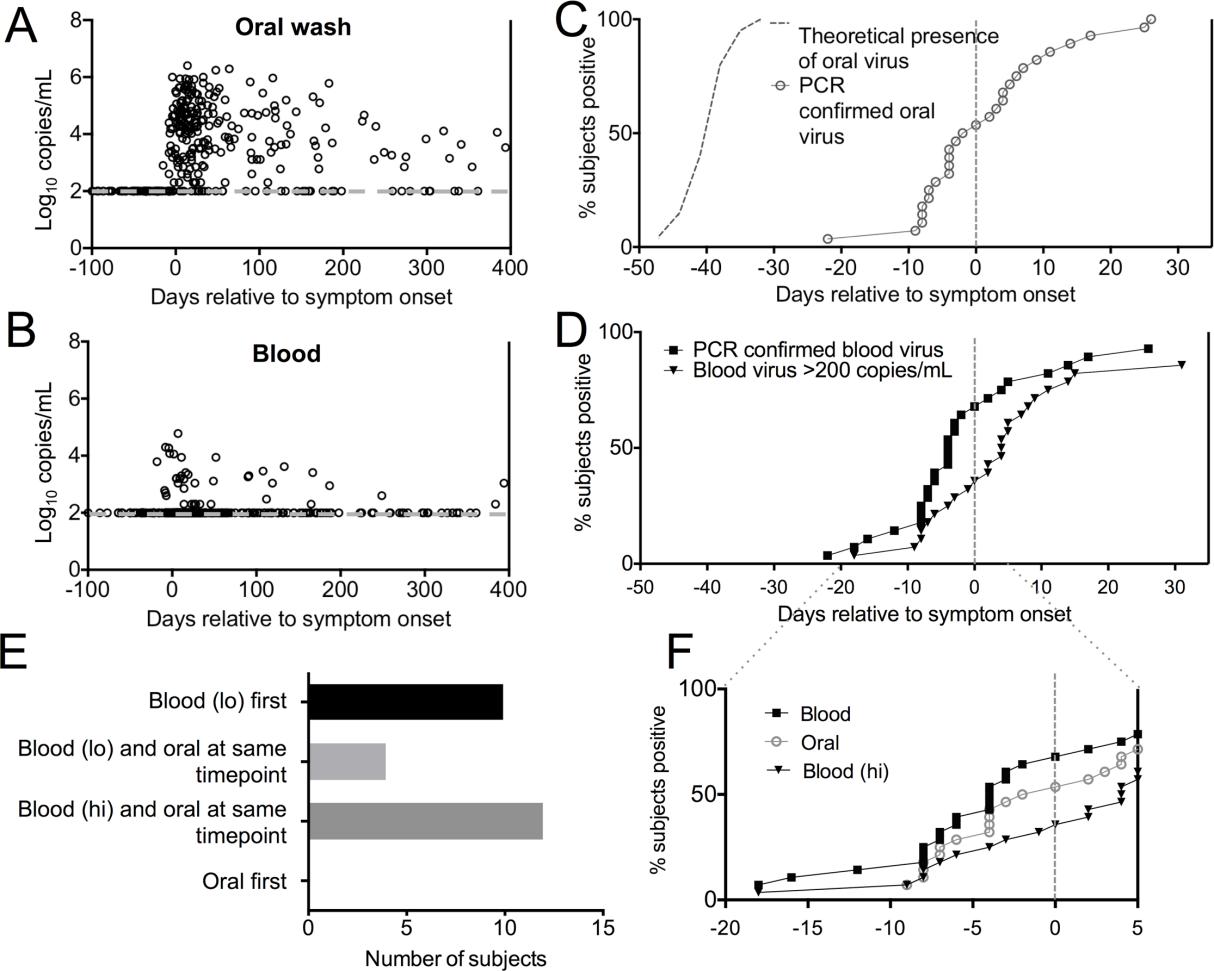
Viral genome detection during the incubation period. Quantitative viral load was determined by qPCR using DNA from oral wash cell pellets (A) or blood (B). Data are expressed as Log_10_ viral genome copies/mL of sample. The dashed gray line represents the limit of detection. (C) and (D) show the time to the first positive measurement for each subject for viral genomes detected in the blood (D) by non-quantitative nested PCR (filled squares) or qPCR (filled inverted triangles), or in the oral cavity (C) by nested or qPCR (same results were obtained with both assays) (open circles). The theoretical presence of virus shown in (C) is the estimated time period in which study participants were initially exposed to oral virus. (E) In sequential samples from the incubation period, subjects were scored for which compartment viral genomes were first detected in: blood by nested PCR (blood (lo)), blood by qPCR (blood (hi)), oral, or a simultaneous positive in both compartments. (F) Shows an inset comparing blood and oral cavity for the time period close to symptom onset. The results for twenty-six subjects who had a sample collected within the first two weeks of symptom onset are shown.

EBV is transmitted through salivary exchange in young adults and infection is established in the oral cavity, both in squamous epithelial tissue and lymphocytes of the Waldeyer’s ring [18]. Very little is known, however, about when and how EBV egresses from tonsillar tissue into the peripheral blood. In order to examine dissemination during the 6 week incubation period more closely we tested for the presence of viral genomes by both quantitative PCR (qPCR) and by a highly sensitive nested PCR. The nested PCR assay gave a much more sensitive but non-quantitative readout of viral presence, whereas the limit of detection for the quantitative PCR assay was 200 copies of EBV per milliliter of whole blood or 40 copies of viral genome per millilter of oral wash. Surprisingly, viral genomes were not detected in the oral cavity by either method until approximately a week prior to symptom onset, at which point large amounts of viral genomes were detected (Fig 1A). These data are expressed at a per subject level as “time to first response” in Fig 1C. Viral genomes were detected in both cells from the oral wash as well as supernatant, suggesting that virus persists in the oral cavity at very low levels for the first 4–5 weeks after transmission, and then exhibits an explosive pattern of replication.

In contrast, viral genomes were detected in peripheral blood as early as 22 days prior to symptom onset, but was only detected via the more sensitive nested PCR assay at this early stage (Fig 1B and 1D). In fact, 10 subjects showed viral genome detection in the blood before the oral cavity when consecutive timepoints were evaluated (Fig 1E). It should be noted that we were able to obtain substantially more DNA from blood cells than from oral wash cells, which could explain why low levels of viral genomes were not detected early in the oral cavity. Nonetheless, we detected dramatically higher viral loads in the oral wash at the time of symptom onset (Fig 1A), suggesting efficient viral detection in oral samples. Virus detection in the blood using the less sensitive qPCR assay was delayed by at least a week (Fig 1F), and viral genomes >200 copies/ml were not found in any individuals until on or after timepoints where high viral loads were detected in the oral cavity (Fig 1E). These data provide the first description of EBV viral dynamics during the incubation period of natural infection in humans, and suggest a scenario where viral replication is self-limiting in the oral cavity for many weeks. Dissemination to the blood occurs during this “quiet period”. Closer to the time of symptom onset, virus replicates rapidly in the oral cavity, and subsequently high viral loads are detected in the blood.

### Gene expression changes in peripheral blood are apparent 1–2 weeks prior to symptom onset

**Fig 2:**
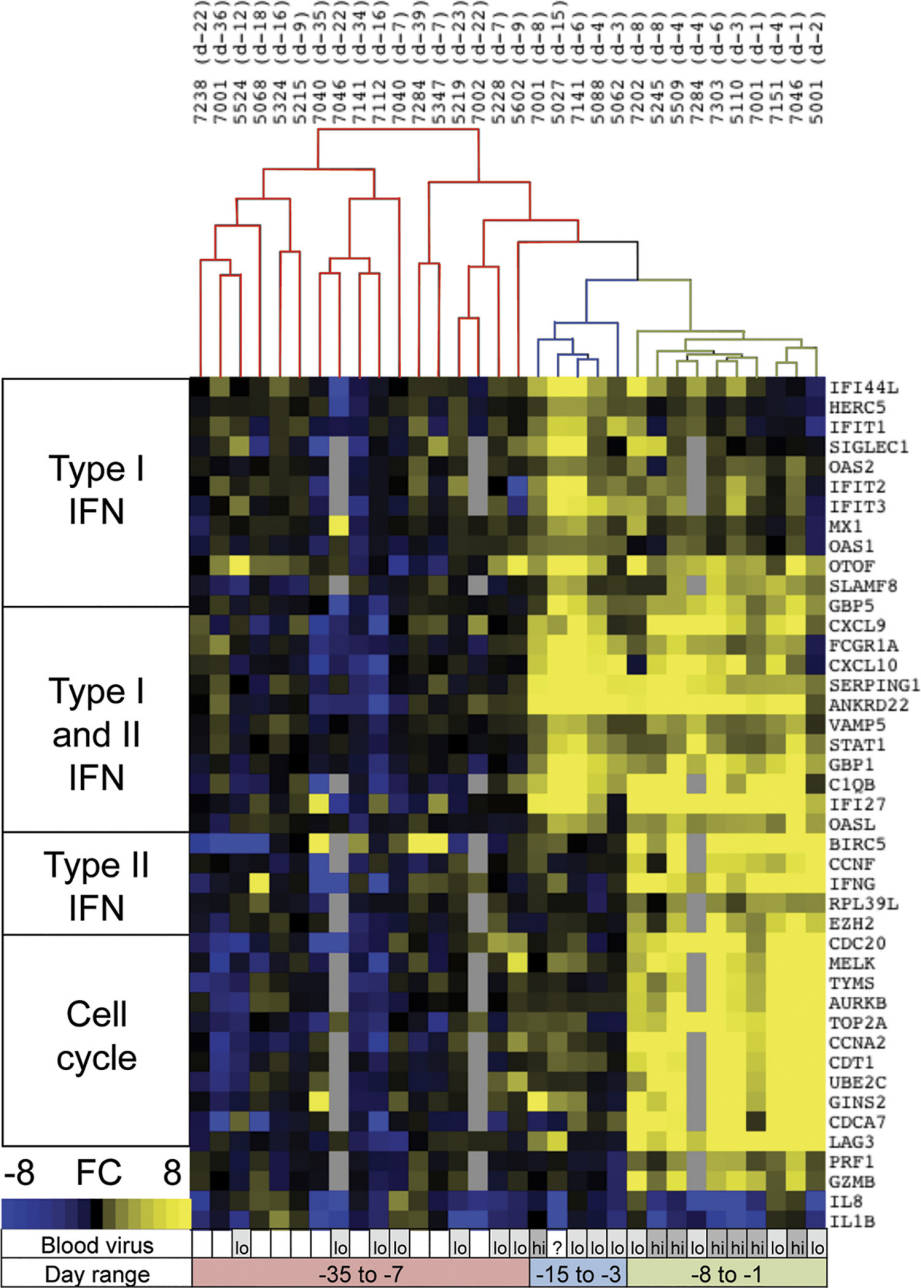
Gene expression signatures during the incubation period showed distinct kinetic patterns. 43 EBV infection signature genes were measured in total PBMC RNA. Functional categorization of the genes is shown at left. Fold change (FC) in expression was calculated compared to pre-infection samples. Heirarchical clustering (above) showed three distinct groupings: no signature (red), type I IFN (blue), and type II IFN/cell cycle (green). Subject numbers and sample collection date are indicated at top. The presence of viral genomes by nested (lo) or qPCR (hi) is noted below, along with the range of sample days (relative to symptom onset) that each signature was observed in.

Previous work from our group revealed that distinct gene expression signatures were present in peripheral blood mononuclear cells early versus late after primary EBV infection [19]. We sought to expand this dataset by examining the additional incubational samples obtained from our most recent cohort. Samples were evaluated by PCR SuperArray consisting of 43 genes representative of gene changes initially observed by microarray [19]. Heirarchical clustering revealed three distinct patterns from incubational samples (Fig 2). Subjects exhibited either no change, a type I interferon (IFN) signature, or a type II IFN/cell cycle signature. These signatures clustered temporally, segregating into these approximate time frames: (i) no change seen from -42 to -7 days prior symptom onset, (ii) a type 1 IFN signature from -15 to -3 days, and (iii) the distinctive type II IFN/cell cycle signature associated with AIM within days of symptom onset. Notably, the type I IFN signature was present when viral genomes were detected only by nested PCR (low viral loads) in 3 out of 4 subjects, although 7 subjects showed no interferon response despite the presence of viral genomes in blood by nested PCR. By ELISA, we detected no substantial (>25 pg/ml) IFNa protein at any timepoints.

### Plasmacytoid dendritic cell numbers in the circulation decrease as viral genomes first become detectable

**Fig 3:**
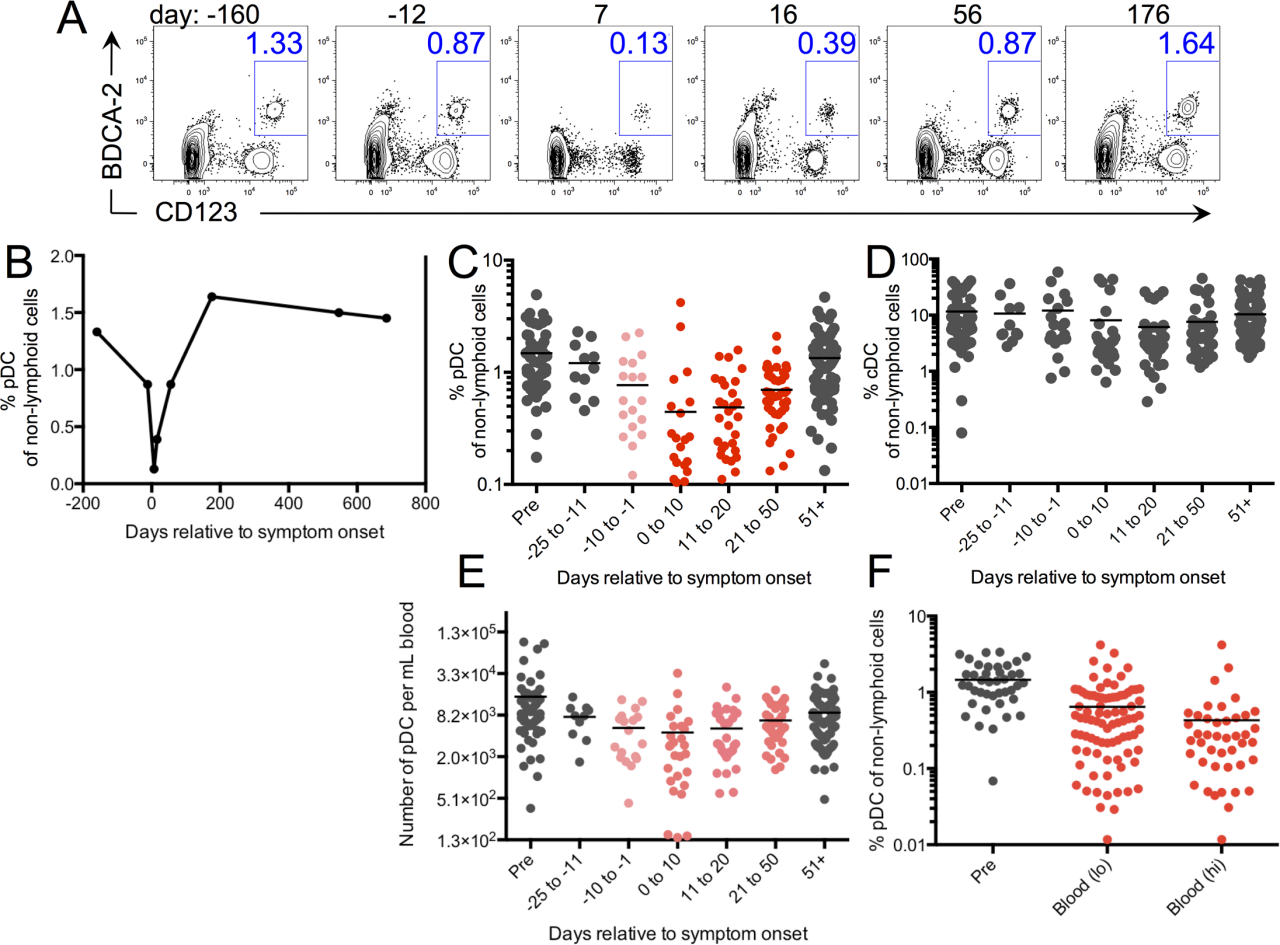
Plasmacytoid DC declined in the circulation during the incubation period and remained depressed through convalescence. (A) Representative flow cytometry plots of pDC frequencies amongst non-lymphoid cells (CD3, CD56, CD14, CD20 negative) from samples collected at multiple timepoints for one subject (5524). (B) The percentage of pDC from 5524 over time. (C) Frequencies of pDC over time are shown for all subjects. (D) Frequencies of conventional DC (cDC) (CD11c^+^, HLA-DR^+^ cells) are shown over time for all subjects. (E) Numbers of pDC per mL of whole blood are shown for all subjects. (F) shows the percentage of pDC in samples where viral genomes were detected in the blood by nested PCR (Blood lo) or qPCR (Blood hi). Statistical analysis was performed using a one-way ANOVA with multiple test comparison. Light pink symbols indicate a significant difference (p<0.05) compared to pre-infection; darker pink symbols (p<0.001); red symbols (p<0.0001). Gray symbols indicate no statistical difference.

Plasmacytoid dendritic cells (pDC) are major producers of type I IFN. Although a robust type I IFN response was observed during the incubation period in some study participants, the gene expression signature was relatively transient. Khanna’s group recently found pDC numbers to be reduced in acute IM patients [20]. Thus, we thus sought to examine pDC numbers during the incubation period. pDC were identified as BDCA-2+ CD123+ cells amongst non-lymphoid cells (CD3, CD20, CD56 and CD14 negative) and were HLA-DR+ and CD11c-. As an example, flow plots are shown for 6 timepoints from subject 5524 (Fig 3A and 3B). Analysis of pDC from all subjects during the incubation period revealed a remarkable decline during the ten-day period leading up to symptom onset (Fig 3C and 3E). A slight decline was observable before that, but was not statistically significant. In contrast, conventional myeloid derived DC (cDC), identified as HLA-DR+ CD11c+ amongst non-lymphoid cells, were not significantly increased or decreased during either the incubation period or the early phase of AIM (Fig 3D). The loss of pDC in circulation was strongly correlated with the presence of viral genomes in peripheral blood (Fig 3F). Interestingly, the reduction in pDC was as profound at timepoints where only low levels of viral genomes were detected as when high levels of viral genomes were detected (Fig 3F), and there was no significant correlation between the number of viral genomes present and the extent of pDC reduction.

### Natural killer cell ratios and phenotype were not affected until onset of AIM

**Fig 4:**
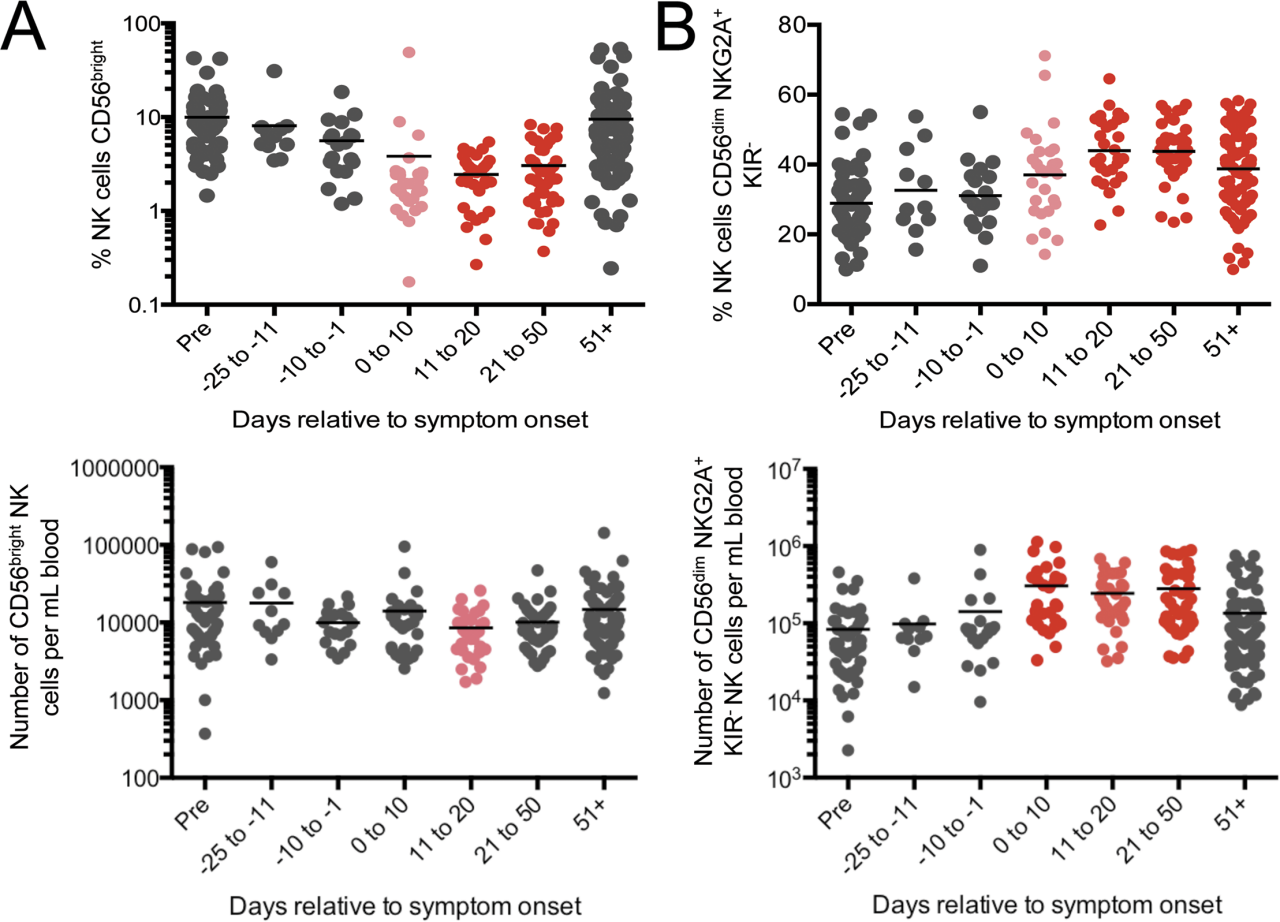
NKG2A^+^ NK cells were expanded during AIM, but not during the incubation period. (A) Percentage and number of NK cells that are CD56^bright^ (immature) decreases during the first 50 days after symptom onset. (B) The percentage and number of NK cells that are CD56^dim^ NKG2A^+^ KIR^-^ increases, and remains elevated. Statistics were performed using a one-way ANOVA with multiple test comparison. Pink symbols indicate a significant difference (p<0.05) compared to pre-infection. Red symbols indicate a significant difference (p<0.0001) compared to pre-infection. Gray symbols indicate no statistical difference.

The importance of NK cells in EBV infection has become increasingly apparent in recent years [21]. Changes in NK cell phenotype during AIM were reported previously, and included a loss of CD56^bright^ “immature” NK cells and a corresponding increase in CD56^dim^ “mature” NK cells [22]. We also observed a reduction in the percentage and number of CD56^bright^ NK cells in this cohort; however, unlike pDC changes, NK cell changes were not apparent until symptom onset (Fig 4A). We further examined a specific CD56^dim^ NKG2A+ KIR- cell subset reported to be expanded during AIM as a consequence of virus-induced proliferation [23]. We observed a similar expansion in our cohort, but it likewise was not detected until symptom onset, and remained elevated for at least 50 days (Fig 4B).

### Polyclonal CD8 T cell activation was detected during the incubation period, but virus specific CD8 T cell expansion was not

**Fig 5:**
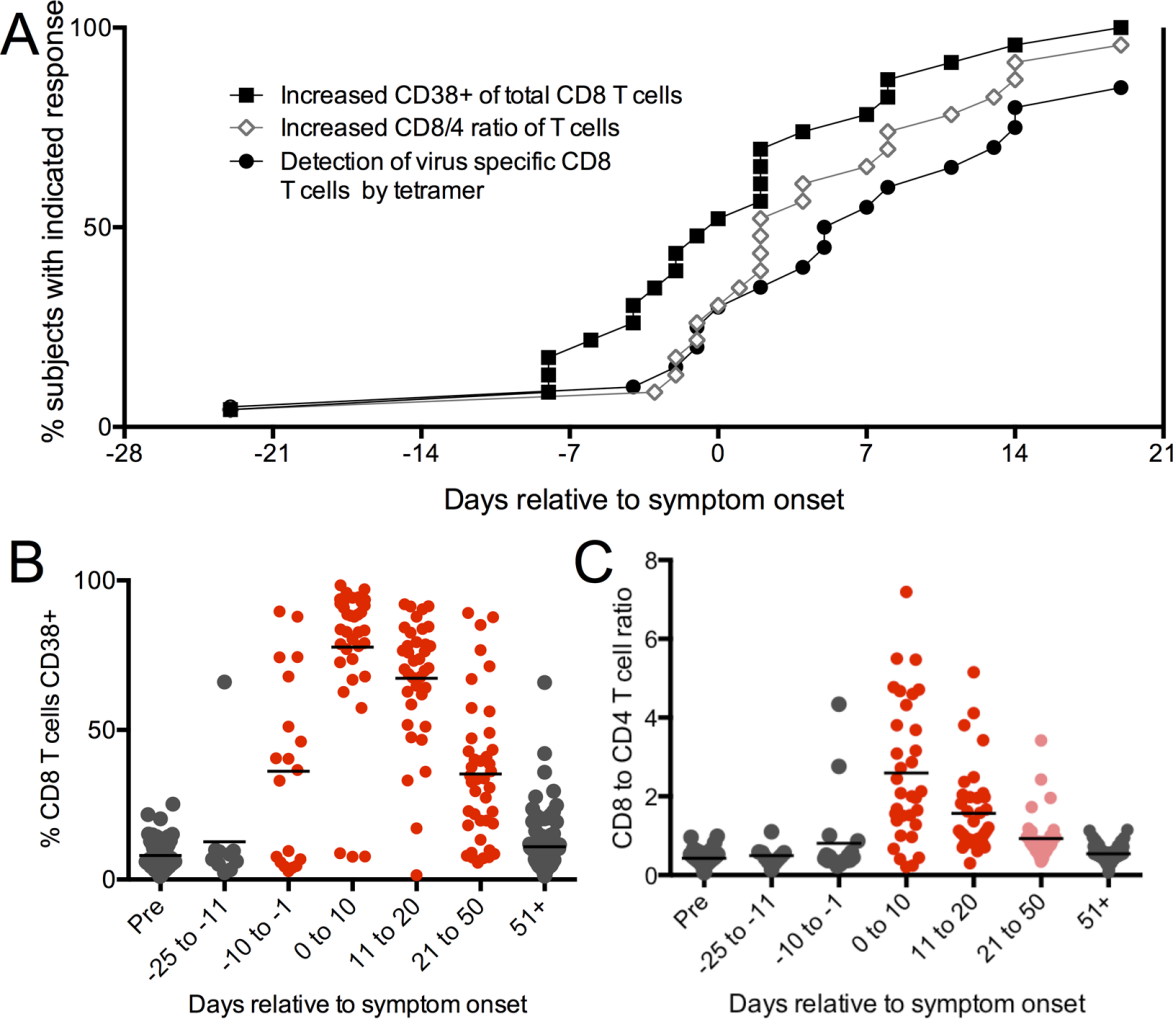
CD8 T cell activation occurred during the incubation period, although not an EBV specific response. (A), Time to first response for three distinct immune parameters is shown: CD38 upregulation on total CD8^+^ T cells (filled squares), an increased CD8 to CD4 T cell ratio (open diamonds), or the presence of EBV tetramer binding CD8^+^ T cells above background (0.4%) (filled circles). (B) Frequency of CD8^+^ T cells expressing CD38 over time. (C) Ratio of CD8^+^ to CD4^+^ T cells over time. Statistics were performed using a one-way ANOVA with multiple test comparison. Pink symbols indicate a significant difference (p<0.05) compared to pre-infection. Red symbols indicate a significant difference (p<0.0001) compared to pre-infection. Gray symbols indicate no statistical difference.

CD8 T cells provide vital immune control of EBV [6], and although their expansion during AIM has been well documented, it is not known when they first become activated during primary infection. Using peptide:MHC I tetramers, we detected no EBV specific CD8 T cell expansion (tetramer binding cells above .05% of CD8 T cells) until timepoints near the onset of symptoms (Figs 5A and S1). Similarly, upregulation of CD11a and downregulation of CD45RA on tetramer binding T cells, indicating antigen experience, were not seen until symptom onset. The expansion of EBV specific T cells was tightly concordant with total CD8 T cell expansion, as reflected by an increased CD8:CD4 ratio (Fig 5A and 5C). Interestingly we detected upregulation of CD38 and granzyme B on total polyclonal CD8 T cells earlier, during the incubation period (Figs 5A, 5B and S1). These features of polyclonal activation correspond kinetically to when a type I IFN response was most strongly represented (Fig 2).

### Foxp3+ CD4 T cell numbers in the circulation decrease after presentation with AIM

**Fig 6:**
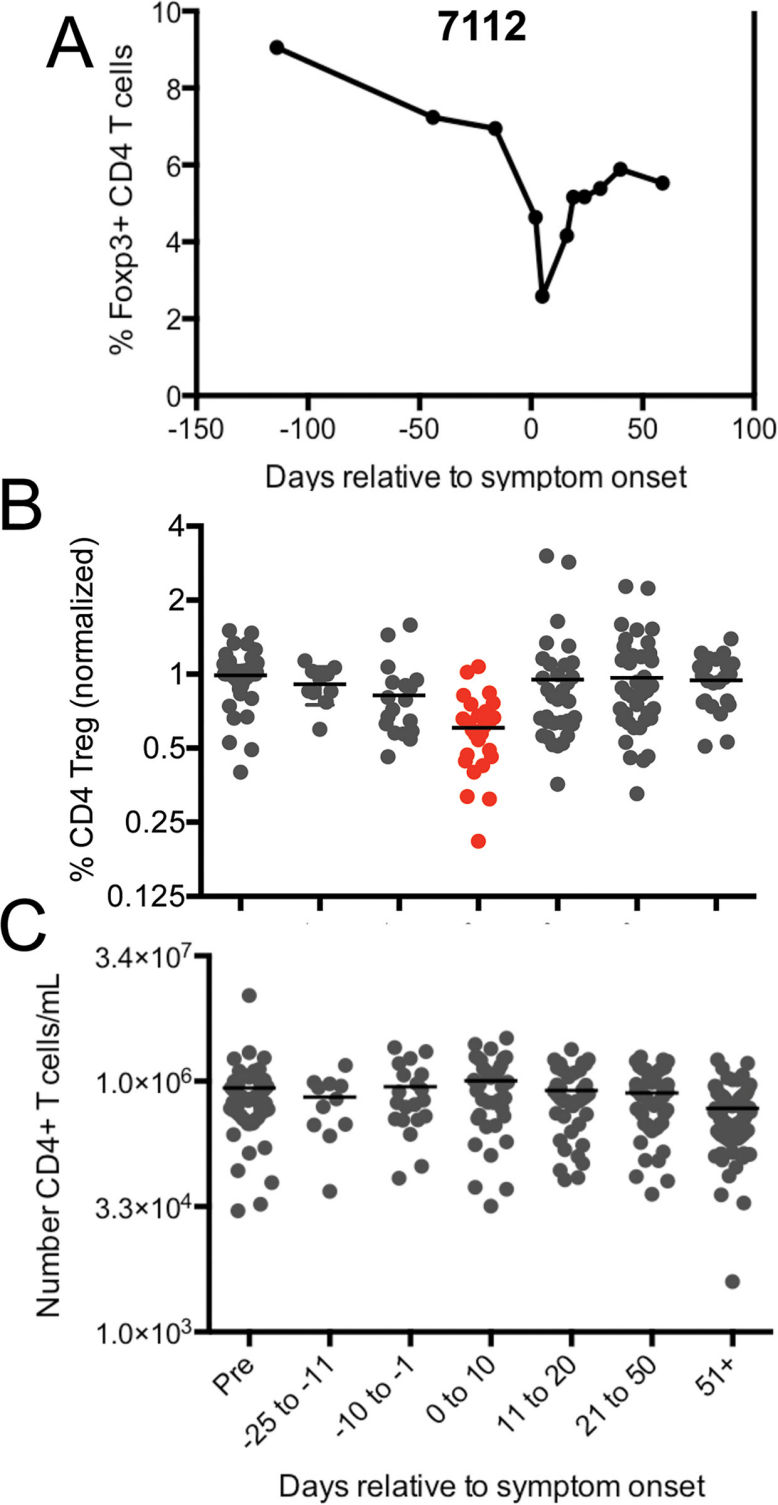
CD4^+^ Foxp3^+^ T cells transiently decline in the circulation at symptom onset during AIM. (A) Frequency of Foxp3^+^CD25^+^ cells amongst total CD4^+^ T cells data plotted over time for a representative individual (subject 7112). (B) Normalized frequency of Foxp3^+^ CD4 T cells over time in all subjects. Foxp3^+^ frequencies were normalized to each subject’s pre-infection baseline due to substantial variation in this population between individuals. (C) Number of CD4+ T cells per mL of whole blood over time. Statistics were performed using a one-way ANOVA with multiple test comparison. Gray values are not statistically different. Red value p<0.0001 compared to pre.

Foxp3+ CD25+ T_reg_ cells, are important for the maintenance of self-tolerance and dampening chronic inflammation. A reduction in the number of circulating CD25^hi^ CD4+ T cells was previously reported in AIM patients [24], but it was unknown when these changes began to manifest and how long they persisted through convalescence. Analysis of individual subjects over time in our study corroborated a decrease in T_reg_ cells at the onset of AIM (Fig 6A and 6B, shown for a representative subject). In all subjects, T_reg_ cells were significantly decreased only during the first ten days of AIM (Fig 6B). Numbers were depressed as well as frequency. The overall number of CD4+ T cells, in contrast, was unchanged (Fig 6C). Although the fate of blood T_reg_ cells during AIM is unknown (e.g. whether they trafficked to tissues or died), previously reported histology of AIM tonsils would argue against local infiltration into tonsils, although the sample size of this study was very small [24].

## Discussion

Our findings have important implications regarding how EBV infection progresses through natural routes in its native host. Despite an oral transmission mode, viral genomes were not detected in the oral cavity in appreciable quantities until subjects had presumably been infected five to six weeks. The lack of detectable viral genomes in oral wash argues against substantial lytic replication within squamous epithelial cells early during infection. Rather, it is consistent with the idea that B cells are a major cell type initially infected in the nasopharyngeum (S2 Fig). EBV efficiently infects B cells, particularly when virus is derived from epithelial sources, which it likely would be during transmission, since virus produced by epithelial cells packages more gp42 into virions than virus produced by B cells [12]. Infected B cells are known to divide and differentiate, replicating the viral genome as an episome along with cellular division [18]. This “vertical” replication would be expected to expand viral load relatively slowly, compared to active viral replication in lytically infected cells. Starting approximately 1 week before symptom onset, viral genomes became detectable at high copy number in the oral wash. It is unclear what event precipitates this sharp increase, but it was not gradual like the decline in viral loads in the oral cavity during latency. It has been postulated that undefined signals may trigger viral reactivation in latently infected B cells [25], which could then lead to high local production of virus and large-scale infection of epithelial cells.

Interestingly, our data would also suggest that infected B cells begin to disseminate into circulation prior to events that precipitate large scale viral production in the oral cavity (S2 Fig). Notably, in 10 subjects we detected low levels of viral genomes in peripheral blood at timepoints prior to detection in the oral cavity. Furthermore, in 7 subjects with low viral genomes present in the blood, there was no type I IFN response detected. This is consistent with the idea that virus is disseminated into circulation via latently infected memory B cells [26] where it goes undetected by the innate immune system. Indeed, infected B cells were shown to transition into a “latency 0” stage that closely resembles resting memory B cells, with altered trafficking patterns [27].

Another point that emerges clearly from these data, is that systemic innate and adaptive immune responses do not occur until viral loads rise relatively late in the incubation period, either in the oral cavity or the blood. The earliest responses detected were a type I interferon response (Fig 2) and upregulation of CD38 on total CD8 T cells (Fig 5), which occurred during the 10 days prior to symptom onset. These two observations may be related, as it was previously shown that type I IFN can upregulate granzyme B in CD8 T cells, independent of activation through the antigen receptor [28]. Indeed, the proportion of CD8 T cells that upregulated CD38 and Granzyme B (>80% in some individuals) at these early time points is too high to be explained by T cells recognizing virus through their antigen receptor, as clonal expansion had not been detected at these time points. Of note is the fact the type I IFN response was relatively transient and not associated with symptoms in any of the study subjects.

An adaptive immune response followed these early events, with expansion of virus specific CD8 T cells, and increased CD8:CD4 ratios rising in all subjects in the first 10 days following symptom onset. IgM responses to EBV viral capsid antigen were also detected in this time frame. As previously reported, IgG responses to VCA developed subsequent to IgM, and IgG responses to EBNA-1 were not maximal until after 3 months. Foxp3+ T regulatory cells were reduced during symptomatic IM as reported previously [24]. A similar reduction is observed in various infections in mice [29, 30], where reduction of effector cell IL-2 in the face of inflammatory cytokines was suggested to be the mechanism [31]. Too little Treg activity can result in immunopathology [31], and we did observe an inverse correlation between Treg percentages during acute infection and disease severity (Spearman r = 0.4871, p = 0.0251) although whether this is causative remains to be explored in EBV.

Curiously, blood NK responses were observed only after symptom onset and not earlier, although NK cells are thought to function early in infections. This result may not be entirely unexpected, as NK cells respond preferentially to lytically rather than latently infected cells [32], and our results suggest that latently infected cells are introduced into circulation prior to lytically infected cells. NK cells are thought to play a protective role in AIM as evidenced by NK cell depletion in humanized mice infected with EBV, which resulted in higher levels of viral DNA in blood [32]. It is possible that NK cells in the tonsil play a critical role in humans, limiting viral spread amongst epithelial cells. Furthermore, NK cells have been hypothesized to play a role in the age dependence of symptomatic primary EBV infection. For example, newborns were reported to have more than twice as many circulating CD56^dim^ NKG2A+ KIR- NK cells than adolescents [23], which could explain why children experience less EBV associated morbidity in comparison with adolescents and young adults. However, it was recently reported that children with asymptomic primary EBV infection have blood viral loads as high as adults with AIM [33], which is not consistent with a model of better NK control of EBV infected PBMC in infants. By closely observing the viral and immune dynamics during natural infection, we offer a new hypothesis on AIM pathogenesis, which proposes that explosive viral replication in the oral cavity creates a situation of exaggerated CD8 T cell response. It may be that children experience less AIM than adults despite ultimately achieving equally high levels of virus in the oral cavity and in blood, because infection in the oral cavity was not initially held in check. This allowed the adaptive immune response to develop by the time blood levels of virus increased. Indeed, memory CD8+ T cells specific for the virus, were observed in asymptomatic children concurrent with high viral loads [33]. Ironically, it may be heightened oral innate immune surveillance in adolescents and adults, compared with children, that puts them at risk for AIM.

A final point of interest in our study was that circulating pDC percentages and numbers were significantly diminished during the viral incubation period. The pDC decrease began during the same period as a type I IFN response and polyclonal CD8 T cell activation were observed—the 10 days prior to symptom onset. Unlike the type I IFN response, which was transient, the pDC reduction was sustained for up to 50 days. The reduction was also observed at all timepoints (except one) that showed the presence of viral genomes, even low levels of viral genomes, and even when a type I IFN response was not present. From this we conclude that viral infection or viral products were responsible for the pDC reduction, but it was unlikely to be mediated by the host’s type I IFN response. This reduction could be related to the dynamics of pDC activation. Evidence in the literature suggests that pDC can mature or leave the circulation into tissues or secondary lymphoid organs during infections [34]. Upon activation, pDC enter a maturation program that can result in progression of pDC into antigen presenting cells [35]; however, we did not see corresponding increase in the MHC class II molecule HLA-DR. Alternatively, reduction of circulating pDC numbers may be related to the BamHI-A rightward frame 1 (BARF1) protein secreted by EBV during lytic replication [36]. BARF1 enhances viral replication and persistence in part by binding to and inhibiting the signaling of colony stimulating factor (M-CSF) [37, 38] an important factor for the survival and maintenance of pDC [39]. It would be interesting to determine if primates infected with a BARF-1 deficient form of lymphocryptovirus show pDC reductions or not. The functional consequences of pDC loss from the blood during primary infection remain to be explored.

An important caveat to this work is that we have been limited to sampling peripheral blood and washings from the oropharynx. It is unknown whether or not we would be able to detect viral DNA or cellular responses to EBV (like a type I interferon response) if we were able to evaluate tonsillar tissue. The possibility that these cells may be sequestered in local tissue cannot be ruled out and remains to be investigated in future studies.

In summary, we report several novel findings about the viral and immune dynamics during the lengthy incubation period of primary EBV infection. These include relatively early dissemination of virus into circulation in a form that does not elicit immune responses. A sharp increase in viral load subsequently occurs in the oral cavity and blood within 10 days of symptom onset. An early type I IFN response during this period is associated with a marked drop in blood pDC numbers and polyclonal CD8 T cell activation, without notable symptoms. Symptom onset coincides with a developing adaptive immune response and a strong type II interferon signature. Severity of illness correlates most strongly with increased CD8 T cell numbers, confirming the notion of AIM as an immunopathologic disease. The sharply increased viral loads that are presumed to drive an exuberant T cell response are already underway prior to symptom onset, providing a potential explanation for the lack of a clear-cut benefit from antiviral drugs in AIM [4]. We also speculate that pre-existing adaptive immunity to EBV would change the dynamics of infection in the oral cavity and thereby prevent IM in adolescents and adults.

## Materials and Methods

### Study design

Samples analyzed here were obtained from two studies: one with a large number of subjects and less frequent sampling (Mono 5) [17] and another with a smaller number of subjects with more frequent sampling (Class of 2016). For the Mono 5 study, healthy undergraduate volunteers from the University of Minnesota were recruited in 2006 and 2007. We screened 546 participants for IgG antibodies against EBV viral capsid antigen (EBV VCA IgG) and EBV nuclear antigen-1 (EBNA-1). Of the 202 eligible EBV-naïve subjects, 143 (71%) were enrolled in the prospective study. Blood and oral washings were collected approximately every 4–8 weeks from enrolled participants during the academic year. Symptoms between visits were reported via an electronic monitoring journal. Subjects with symptoms consistent with acute primary EBV infection were asked for an additional visit which included a physical exam, laboratory-confirmation of primary EBV infection via heterophile, EBV-specific serology, and viral titer in the oral cavity or blood. Primary EBV infection was defined as a positive EBV antibody test and the presence of EBV DNA in the blood and/or oral cavity of a subject who was previously negative for both EBV antibodies and EBV DNA. All participants were monitored with follow-up visits. The Class of 2016 study was similar to the above except blood and/or oral washings were collected approximately every 2 weeks. We screened 279 participants and 87 EBV-naïve subjects were enrolled in 2012, 16 of whom experienced primary EBV infection during their 9 month freshman year.

### Ethics statement

All participants gave written informed consent and the University of Minnesota Institutional Review Board approved all protocols used.

### Samples collection and handling

Subjects gave oral wash samples by gargling with 22mL of normal saline. Suspended oral cells were separated from supernatant by centrifugation and frozen in two aliquots at -80°C. Four 1ml aliquots of supernatant were saved and frozen at -80°C. Peripheral blood was obtained via venipuncture into EDTA Vacutainer tubes (Fisher Scientific). Blood Peripheral blood mononuclear cells (PBMCs) were isolated by Accuspin System-Histopaque-1077 (Sigma-Aldrich) density gradient centrifugation per manufacturer’s instructions. PBMC were divided into 1x10^7 cells/mL aliquots in a 90% FBS and 10% dimethylsulfoxide solution to prevent cell damage (Sigma-Aldrich). Vials were placed inside Mr. Frosty freezing containers (Thermo Scientific) and frozen at -80°C per the manufacturer’s instructions, then transferred to liquid nitrogen for long term storage. Cells were rapidly thawed in a 37°C water bath, diluted to 10ml in RPNK media supplemented with 50U/ml benzonase (Novagen) (RPNK media: RPMI 1640 (Cellgro) supplemented with 10% FBS (Atlanta Biologicals), 2% Penicillin—Streptomycin (5000U/ml, 5000μg/ml respectively, GIBCO, Invitrogen) and 1% L-glutamine (29.2mg/ml, GIBCO)). Cells were then counted using a hemocytometer and divided into separate fractions for flow cytometry, RNA processing, and/or DNA processing.

### Flow cytometry staining and analysis

Multiple time points were chosen from subjects who gave a blood sample during the incubation period. 1 to 2 x10^6 PBMCs from each of these time points were used in each stain. The following antibodies were used to identify relevant surface and intracellular markers: CD3 (UCHT1), CD4 (RPA-T4), CD11a (HI111), CD20 (2H7), CD123 (6H6), PD-1 (MIH4), CD25 (BC96), Foxp3 (PCH101) (eBioscience); CD45RA (HI100), CD38 (HIT2), CD16 (3G8), CD57 (HCD57), CD14 (M5E2), CD19 (HIB19), BDCA-2 (201A) (BioLegend); CD56 (NCAM16.2), HLA-DR (G46-6), CD11c (B-ly6) (Becton Dickinson); NKG2A (Z199), KIR2DL1/2DS1 (EB6B), KIR2DL2/2DL3/2DS2 (GL183) (Beckman Coulter); CD8 (3B5) (Invitrogen); KIR3DL1 (DX9), KIR3DL2 (539304) (R&D systems). Intracellular granzyme B staining was performed using the Cytofix/Cytoperm kit per the manufacturer’s instructions (BD). Intranuclear Foxp3 staining was performed using the Foxp3 / Transcription Factor Staining Buffer Set per the manufacturer’s instructions (eBioscience). All samples were acquired using an LSR II (BD) and analyzed with FlowJo software (TreeStar). During analysis, lymphocyte frequencies were normalized to the absolute number of lymphocytes in each sample.

### RNA and DNA extraction

For each sample, 1–2 x 10^6^ PMBCs were used for each RNA extraction. Cells were first homogenized using QIAshredder columns (Qiagen) per the manufacturer’s instructions. RNA was then extracted using RNeasy kit (Qiagen) with on-column DNase step (Qiagen) per the manufacturer’s instructions. RNA was then quantified using a Nanodrop 2000/2000c spectrophotometer (Thermo Scientific) and kept frozen at -80°C. DNA extractions were performed with the Qiagen QIAmp Blood Mini kit per the manufacture’s instructions, using either 200 μL of whole blood or 5x10^6 PBMC.

### Peptide MHC class I tetramers reagents

An EBV BMLF1259–267 (GLCTLVAML)-A*0201 tetramer reagent was purchased from ProImmun. Other biotinylated MHC-peptide monomers were obtained from the National Institutes of Health (NIH) tetramer facility: EBV BRLF1109–117 (YVLDHLIVV)-A*0201, EBV BRLF1147–155 (RVRAYTYSK)-A*03, EBV BZLF1190–197 (RAKFKQLL)-B*08, EBV EBNA3A325–333 (FLRGRAYGL)-B*08, EBNA3A379–387 (RPPIFIRRL)-B*07, and EBNA3A603–611 (RLRAEAQVK)-A*03. Before use, APC-streptavidin (Invitrogen) was added to monomers at a 4:1 molar ratio overnight in the dark at 4°C to generate fluorescent pMHCI tetramer complexes. All tetramers were stored in the dark at 4°C.

### cDNA synthesis

cDNA was generated with 100ng of starting RNA using the SuperScript III Platinum Two-Step qRT-PCR Kit (Invitrogen) per the manufacturer’s instructions. Samples were stored at -20°C.

### Quantitative PCR

Standard quantitative PCR was performed with FastStart Universal SYBR Green Master (Rox) (Roche) per the manufacture’s instructions. Additional data were generated with precoated “SuperArray” PCR plates. 43 genes were selected for analysis by PCR from a larger list of changed genes in IM subjects and other acute viral infections that comprised relevant functional groupings as assessed by Ingenuity Pathway Analysis as previously described [19]. 384-well SuperArray plates pre-coated with primers for the desired 43 genes plus 5 controls were obtained as a custom order from SABiosciences. 10 μL of cDNA per subject was used with RT2 Real-time SYBR green/Rox PCR master mix (SABiosciences) for qRT-PCR analysis. Products were detected using an ABI Prism 7900HT Sequence Detection System (Applied Biosystems). The genes *ACTB*, *B2M*, and *RPL13A* were used as housekeeping genes during the calculation of fold changes. Fold changes were calculated as: 2^(Δ Acute Housekeeping Control–Baseline Housekeeping Control)/2^(Δ Acute Gene of Interest–Baseline Gene of Interest).

### Heatmap generation

Fold change values obtained by quantitative PCR were imported into the open source program Cluster 3.0, [40] which clustered the genes hierarchically using a Pearson non-averaged correlation and average linkage. Heatmaps were then visualized using the program Java Treeview. [41]

### Nested PCR

PCR was performed with the HotStarTaq master mix kit (Qiagen) per the manufacture’s instructions. Primers specific for *EBNA1* [42] were used (outer-F 5’-GTA GAA GGC CAT TTT TCC AC-3’; outer-R 5’-CTC CAT CGT CAA AGC TGC A-3’; inner-F 5’-AGA TGA CCC AGG AGA AGG CCC AAG C-3’; inner-R 5’-CAA AGG GGA GAC GAC TCA ATG GTG T-5’).

## Supporting Information

S1 Fig T cell analysis. **(**A) The top two rows show binding of MHC Class I EBV tetramers (pools of 7 lytic/latent antigen/HLA tetramers prepared with APC-streptavidin) to CD8^+^ T cells (top row) or CD4^+^ T cells (control, second row) at the indicated time points relative to symptom onset in subject 7001. The 3^rd^ and 5^th^ rows show plots gated on tetramer^+^ CD8^+^ T cells, showing expression of memory markers (CD45RA and CD11a, 3^rd^ row) or activation markers (CD38 and granzyme B, 5^th^ row). The 4^th^ and 6^th^ rows show expression of memory (4^th^ row) or activation markers (6^th^ row) on total CD8^+^ T cells. (B) Frequency of MHC Class I EBV tetramers (pools of 7 lytic/latent antigen/HLA tetramers) binding CD8 T cells over time.
(TIF)

S2 Fig Model of events during primary infection. Sharing of oral secretion with an infected individual introduces viral particles into the oral cavity. Virus infects rare B cells near the squamous epithelial layer in the tonsil (1). These cells then become transformed entering latency III (2). At this point, infected cells contain a single copy of the viral genome (denoted by light purple), which is replicated as the transformed B cell divides (so called “vertical transmissioin”) (3). Only after transitioning to resting memory-like latency 0 cells, do virally infected B cells disseminate to peripheral blood (4). As yet unknown events trigger viral lytic infection amongst B cells in the oral cavity (5), Lytically infected cells (denoted by dark purple) produce sufficient quantities of virus to infect epithelial cells (6). Epithelial cell derived virus is then shed into the saliva and adjacent tissue, where it initiates a new round of B cell infection (7), and amplifies the response. We hypothesize that AIM could result from steps 1–4 if the transmission inoculum is high enough. But in most people, AIM requires additional rounds of amplification, resulting in a lengthy incubation period. The viral particles produced by B cells (purple) versus epithelial cells (pink) are distinct in their surface glycoprotein composition.
(TIF)
