# Supplementary figures and images for: The Incubation Period of Primary Epstein-Barr Virus Infection: Viral Dynamics and Immunologic Events
Source: PLoS Pathog. 2015 Dec 1;11(12):e1005286. doi: 10.1371/journal.ppat.1005286 (PMC4666617; doi:10.1371/journal.ppat.1005286)

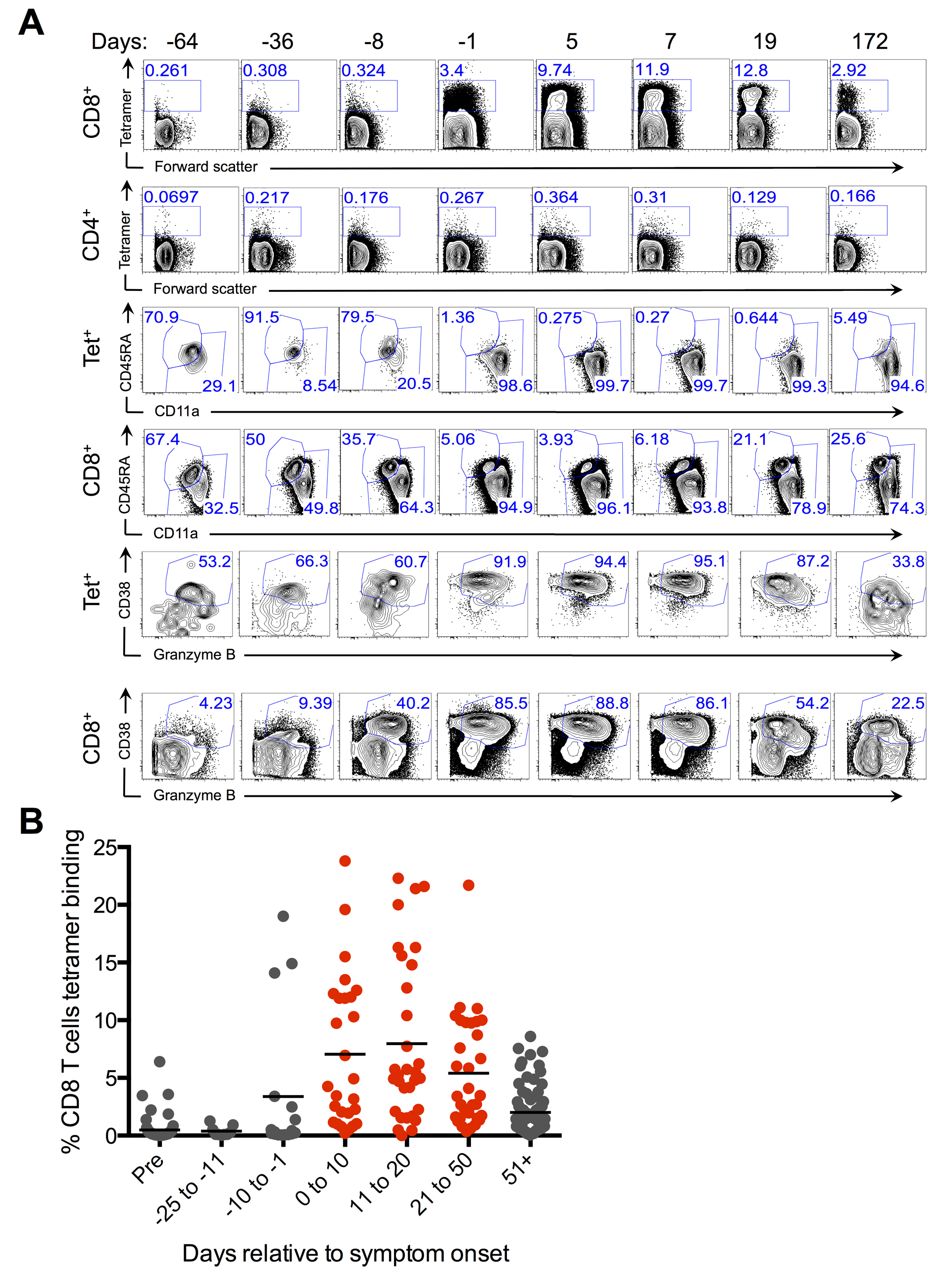

Supplement: S1 Fig — (A) The top two rows show binding of MHC Class I EBV tetramers (pools of 7 lytic/latent antigen/HLA tetramers prepared with APC-streptavidin) to CD8+ T cells (top row) or CD4+ T cells (control, second row) at the indicated time points relative to symptom onset in subject 7001. The 3rd and 5th rows show plots gated on tetramer+ CD8+ T cells, showing expression of memory markers (CD45RA and CD11a, 3rd row) or activation markers (CD38 and granzyme B, 5th row). The 4th and 6th rows show expression of memory (4th row) or activation markers (6th row) on total CD8+ T cells. (B) Frequency of MHC Class I EBV tetramers (pools of 7 lytic/latent antigen/HLA tetramers) binding CD8 T cells over time. (TIF) [file ppat.1005286.s001.tif]

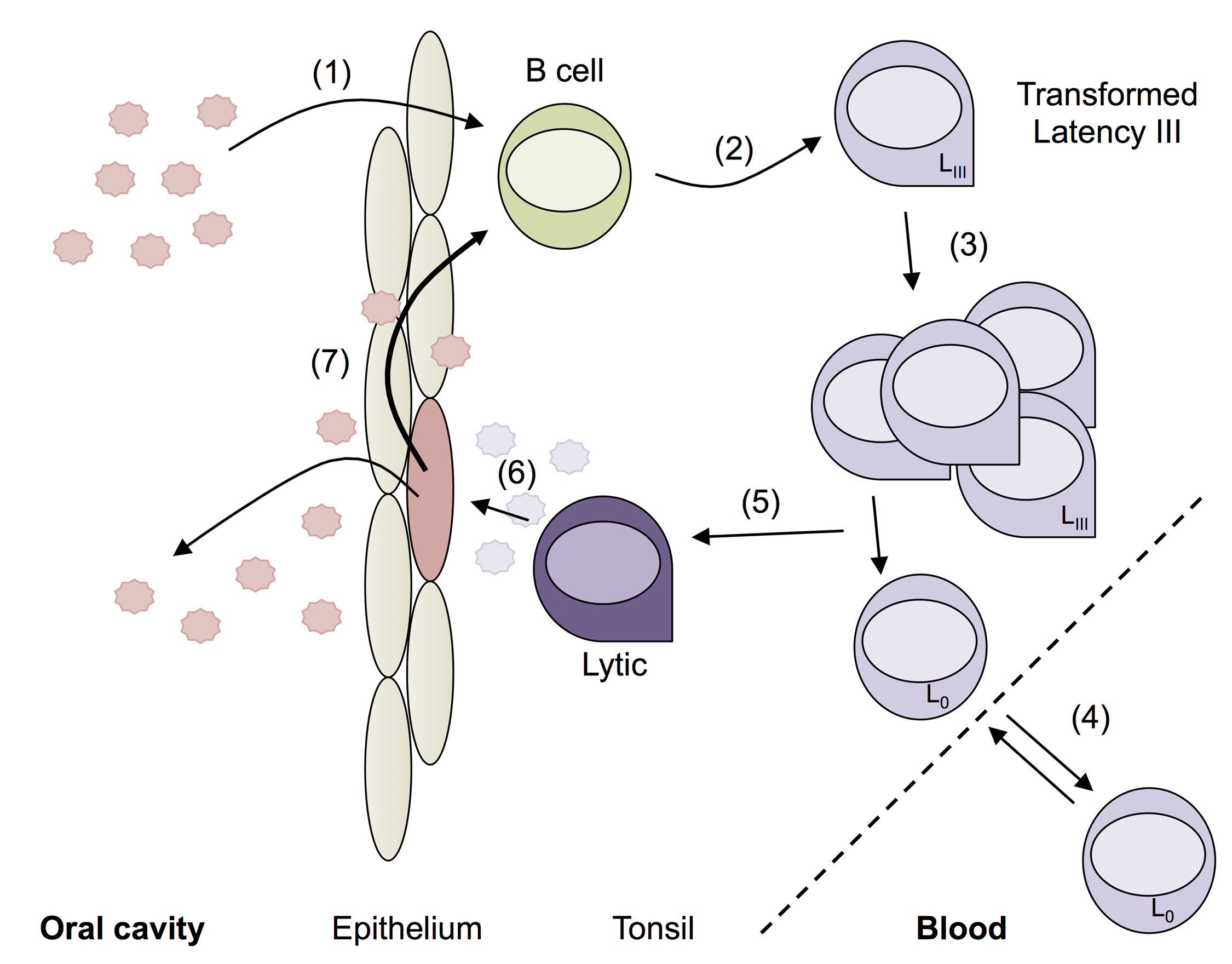

Supplement: S2 Fig — Sharing of oral secretion with an infected individual introduces viral particles into the oral cavity. Virus infects rare B cells near the squamous epithelial layer in the tonsil (1). These cells then become transformed entering latency III (2). At this point, infected cells contain a single copy of the viral genome (denoted by light purple), which is replicated as the transformed B cell divides (so called “vertical transmissioin”) (3). Only after transitioning to resting memory-like latency 0 cells, do virally infected B cells disseminate to peripheral blood (4). As yet unknown events trigger viral lytic infection amongst B cells in the oral cavity (5), Lytically infected cells (denoted by dark purple) produce sufficient quantities of virus to infect epithelial cells (6). Epithelial cell derived virus is then shed into the saliva and adjacent tissue, where it initiates a new round of B cell infection (7), and amplifies the response. We hypothesize that AIM could result from steps 1–4 if the transmission inoculum is high enough. But in most people, AIM requires additional rounds of amplification, resulting in a lengthy incubation period. The viral particles produced by B cells (purple) versus epithelial cells (pink) are distinct in their surface glycoprotein composition. (TIF) [file ppat.1005286.s002.tif]
